# Deep learning in head & neck cancer outcome prediction

**DOI:** 10.1038/s41598-019-39206-1

**Published:** 2019-02-26

**Authors:** André Diamant, Avishek Chatterjee, Martin Vallières, George Shenouda, Jan Seuntjens

**Affiliations:** Medical Physics Unit, McGill University and Cedars Cancer Center, 1001 Décarie Blvd, Montréal, QC H4A 3J1 Canada

## Abstract

Traditional radiomics involves the extraction of quantitative texture features from medical images in an attempt to determine correlations with clinical endpoints. We hypothesize that convolutional neural networks (CNNs) could enhance the performance of traditional radiomics, by detecting image patterns that may not be covered by a traditional radiomic framework. We test this hypothesis by training a CNN to predict treatment outcomes of patients with head and neck squamous cell carcinoma, based solely on their pre-treatment computed tomography image. The training (194 patients) and validation sets (106 patients), which are mutually independent and include 4 institutions, come from The Cancer Imaging Archive. When compared to a traditional radiomic framework applied to the same patient cohort, our method results in a AUC of 0.88 in predicting distant metastasis. When combining our model with the previous model, the AUC improves to 0.92. Our framework yields models that are shown to explicitly recognize traditional radiomic features, be directly visualized and perform accurate outcome prediction.

## Introduction

Radiation therapy is often used (74%)^1^ to treat head and neck (H&N) cancers, a group of neoplasms originating from the squamous cells that line the mucosal surfaces of the oral cavity, paranasal sinuses, pharynx or larynx. Although loco-regional control of most H&N cancers is reasonably good (≈90%)^2^, long-term survival can be quite poor (5-year survival rates as low as 50%)^2^, in large part due to the development of distant metastasis or second primary cancers^3,4^. Thus, the development of a model capable of identifying potential high-risk patients prior to treatment is critical. With such a model, a better-informed decision could be made regarding patient risk stratification. A high-risk patient could be assigned a more aggressive treatment regimen, potentially improving their outcome. Similarly, a low-risk patient could receive a more conservative treatment, delivering less radiation in order to reduce the chance of harmful side effects, such as hormonal disorders, tismus, xerostomia or dental disease^5^. The primary focus of this work is to build a model that is capable of discerning high-risk H&N cancer patients prior to their treatment using solely their computed tomography (CT) image.

Machine learning has played an increasingly prominent role over the past few decades in nearly every aspect of the STEM (science, technology, engineering and medicine) fields^6,7^. Recently, deep learning, a sub-field of machine learning, has risen to the forefront of the artificial intelligence community^8^. One of the most popular deep learning tools is the convolutional neural network (CNN), a type of algorithm inspired by the biological neural networks within the animal visual cortex. CNNs consist of sequential *layers* which contain increasingly complex representations of data, eventually resulting in a classification of the input data^9,10^. In particular, they are very effective at analyzing images and have achieved enormous success in numerous computer vision tasks, such as object detection, semantic segmentation, object classification and CADx (computer-aided diagnosis)^11–19^.

Radiomics is the study of “image biomarkers” - the characterization of tumor phenotypes via the extraction of data from all types of medical images^20^. In the past few years, it has been extensively deployed for outcome prediction, among other applications^21–28^. For image-based outcome prediction, there are three common approaches. The first is the use of handcrafted features^29^ which are directly extracted from the medical images. Often, these features are then fed into a machine learning algorithm for outcome prediction (e.g., random forest, support vector machine)^21–28^. We refer to this as “traditional radiomics”. The second approach uses the outputs of the deeper layers in a CNN (often the final or penultimate fully connected layer) as “deep features”. Similar to the first approach, these “deep features” are then fed into a secondary machine learning algorithm for outcome prediction^17,18,30–32^. The third approach employs transfer learning to fine-tune the weights of a pre-existing network to predict outcomes^9,10^. Our methodology represents a novel fourth approach in that we use a single end-to-end CNN trained *de novo* (with no secondary machine learning algorithms) to predict oncological outcomes. To our knowledge, this is something that has not been successfully attempted in this context. This study will be specifically benchmarked against a previous study on the same data by Vallières *et al*.^25^ which correlated a number of radiomic features from pre-treatment pre-segmented CT images with the outcome of H&N cancer patients. We use a novel deep CNN framework on the same cohort of patients, improving on a number of metrics, both quantitative and qualitative; detailed comparisons are made throughout this report. In the benchmark study, the most predictive combination of radiomic features related with distant metastasis involved *LRHGE*_*GLRLM*_ (long run high grey level emphasis of the grey level run length matrix), *ZSV*_*GLSZM*_ (zone size variance of the grey level size zone matrix) and *ZSN*_*GLSZM*_ (zone size non-uniformity of the grey level size zone matrix)^25,29^. We show that our network is capable of directly recognizing these radiomic features without having any prior information regarding their mathematical definition. While CNNs can be used for image segmentation^11,12,16^, our methodology functions on pre-segmented tumor volumes, both to stay consistent with the benchmark study^25^ and to simplify the task at hand.

One of our primary hypotheses is that a carefully trained CNN could learn the ability to recognize radiomic features. Typically, when attempting to apply a CNN framework to an unexplored image dataset that is of limited size, one uses a methodology called *transfer learning*. In transfer learning, a network that has already been trained and evaluated on another (much-larger) dataset is used as a starting point, and subsequently fine-tuned for the dataset of interest^9,10^. This often results in excellent performance^33^. As an example, ImageNet^34^ is a database of over 1 million RGB images which programmers compete on in an attempt to accurately classify the images into 1 of over 20,000 categories (e.g., dog, cat, plane, car, bench). Transfer learning was used to teach a top-performing network (Google’s Inception-v3^35^) to classify dogs into one of eleven breeds (e.g., bulldog, dachshund) with 96% accuracy^36^. The success is largely because of the *similarity* in the features that distinguish objects and dog breeds, features such as sharp edges or color gradients. In accordance with our goal of training a CNN that can recognize radiomic features, employing a transfer learning approach is possibly less successful. Furthermore, if an adequately sized dataset is available for the relevant classification task, it may not be necessary to use transfer learning as a methodology. Since medical images look substantially different from the everyday world to the human eye, and we have a dataset of 300 patients at our disposal, we decided to explore training a network *de novo* using gray-scale CT images. A comparison to a more traditional transfer learning approach is included in the Supplementary Information to quantitatively evaluate this hypothesis.

The novel contributions of this work are three-fold. Firstly, we develop a deep CNN-based framework capable of *accurately predicting H&N cancer treatment outcomes based solely on a patient’s pre-treatment CT image*. Secondly, the framework is an externally validated medical gray-scale end-to-end CNN built *de novo*, rather than using transfer learning. Finally, the CNN is shown to *explicitly recognize previously engineered radiomic features with proven predictive power*^25^ *on a benchmark study*, and is shown to complement their performance in a number of qualitative and quantitative ways which will be discussed throughout this report.

## Results

### Benchmark study comparison

The testing set of 106 patients used in the benchmark study^25^ is used as an independent validation set in this study. The training set used was identical to that of the benchmark study. The results obtained from our CNN framework are shown alongside the results of the compared study^25^ in Tables 1 and 2. The training and evaluation was done on the *central* tumor slice, which was defined as the slice with the maximum number of tumor pixels within a patient’s entire set of CT images. This is in contrast to the benchmark study, where the model was trained and evaluated on the *entire* tumor volume. To evaluate the model’s robustness with respect to the precise choice of tumor slice, the same network was also trained and evaluated on the slice directly above (superior) and below (inferior) the central slice. In summary, the most powerful network resulted in an area under the receiver operating characteristic curve (AUC) of 0.88 when predicting distant metastasis, comparable to the benchmark result. The most improved network, trained to predict loco-regional failure, had an AUC of 0.65, a substantial improvement over the prior study which was unable to find *any* predictive radiomic features. An AUC of 0.70 was found when predicting overall survival, comparable to the benchmark result. These improvements will have to be further verified by using an additional independent testing set on which the CNN is applied without any change to the hyper-parameters. The precise choice of the evaluation slice did not have a significant impact on the results, as shown in Table 1. It is noted that this study calculated specificity and sensitivity based on an optimized threshold, while the benchmark study^25^ performed imbalance adjustments and used a threshold of 0.5. Additionally, using the same logistic regression methodology described in the benchmark study^25^, we combined the final output score of our DM CNN model with the three aforementioned features used in the DM model of the benchmark study. The DeLong test was used to assess whether the combined model resulted in a statistically significant change in the AUC^37^. The new four-feature model had an AUC of 0.92 in the validation set (*p*-value of 0.04 when compared to the benchmark model, *p*-value of 0.12 when compared to the CNN model). This combination approach could not be implemented for the other outcomes, due to the traditional radiomics model’s inability to find strong individual features.

**Table 1:** Validation set AUC results compared to Vallières *et al’s*^25^ testing set results on the same patient cohort.

	AUC (Area under the curve)				Combined model AUC
	Central slice	Superior slice	Inferior slice	Vallières *et al*.^25^	
Distant metastasis (DM)	0.88	0.88	0.88	0.86	0.92
Loco-regional failure (LRF)	0.65	0.63	0.64	0.50	—
Overall survival (OS)	0.70	0.68	0.67	0.65	—

Robustness was evaluated by training and evaluating networks on the center slice, the inferior slice and the superior slice. Final column represents a combined model utilizing the CNN score and the traditional features from Vallières *et al*.^25^. The combined model was only implemented for DM, due to the traditional radiomics model’s inability to find strong individual features for the other two outcomes.

**Table 2:** Validation set results compared to Vallières *et al.*’*s*^25^ testing set results on the same patient cohort.

	Specificity		Sensitivity		Balanced Accuracy	
	Present study	Vallières *et al*.^25^	Present study	Vallières *et al*.^25^	Present study	Vallières *et al*.^25^
DM	0.89	0.77	0.86	0.79	88%	77%
LRF	0.67	0.61	0.65	0.39	66%	58%
OS	0.67	0.67	0.68	0.55	68%	62%

Balanced accuracy is defined as the average of the specificity and sensitivity. It is noted that this study calculated specificity and sensitivity based on thresholds optimized in the training set, while the benchmark study^25^ performed imbalance adjustments during training and then used a single probability threshold of 0.5 in the testing phase. DM: Distant metastasis; LRF: Loco-regional failure; OS: Overall survival.

### Cross validation

To better assess the stability of our results, we performed 5-fold cross validation on the entire set of 300 DM images. No changes to the hyper-parameters were made between any of the folds and thus remained identical to the hyper-parameters used in the comparison presented above. The mean AUC was found to be 0.85 (range: 0.80 to 0.88). It is noted that the 5-fold cross validation results should not be directly compared to the results of the benchmark study^25^ due to the differing data partitioning scheme.

### Visualization of results

**Figure 1:**
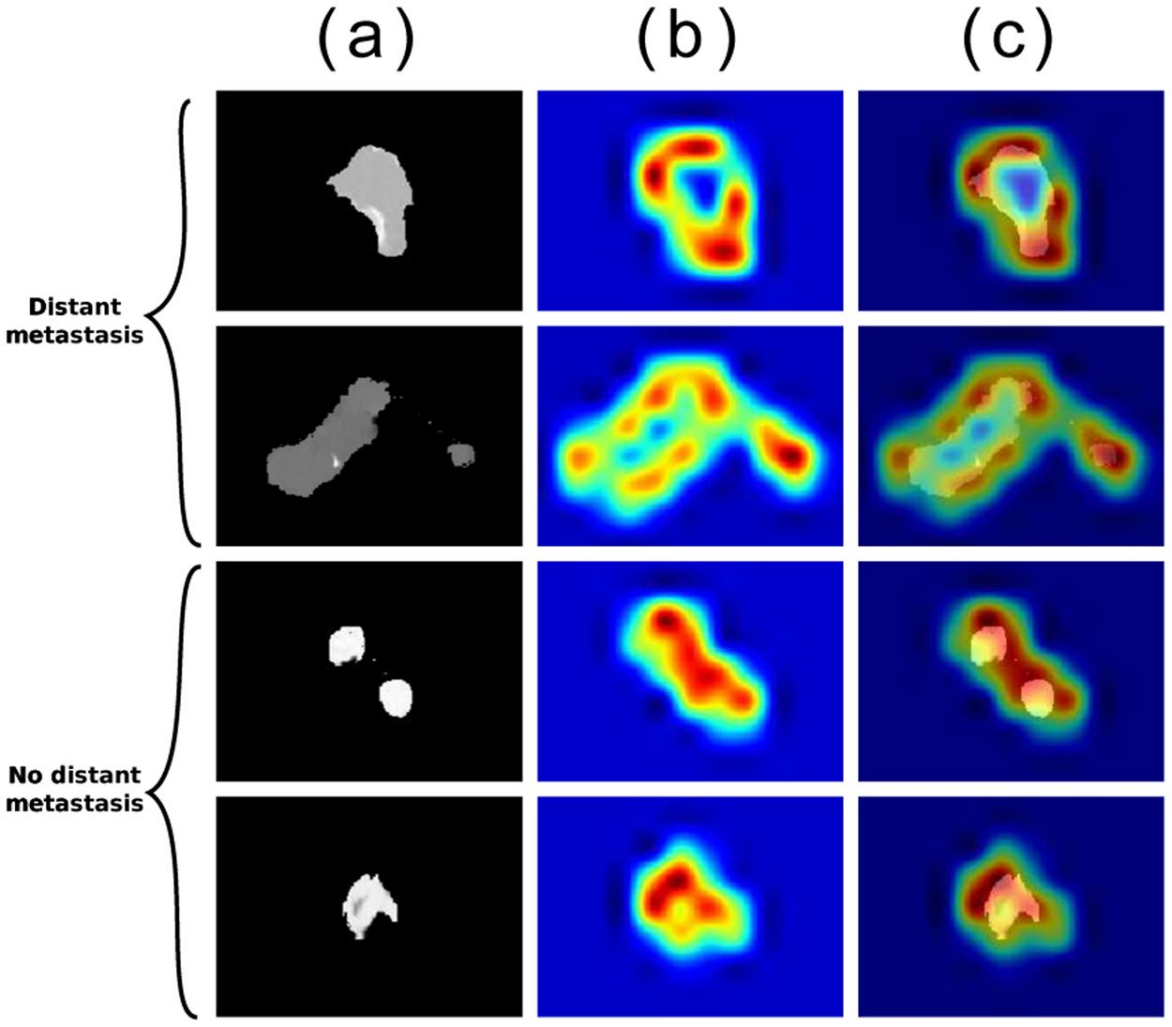
Montage of tumors and gradient class activation maps (Grad-CAM): First two rows represent patients who developed distant metastasis (DM). Last two rows represent patients who did not develop DM. (**a**) Raw image input into the model (zoomed in for visualization purposes). Note that tumor segmentation is performed prior to being input into the model. (**b**) Gradient class activation map (Grad-CAM^39^) of the penultimate convolutional block, red represents a region more significant to the designated classification. (**c**) Image merge of the first two columns.

There are a number of visualization tools that we can use to better understand the behaviour of the CNN, many of which are facilitated by the Keras-*vis* toolbox^38^. All visualization examples in this section represent the highest performing network (i.e., trained on the central tumor slice to predict distant metastasis). Figure 1 represents a montage of four patient CTs, two of whom developed DM (top), and two who did not (bottom). These particular CT images are chosen to represent the diversity in features perceivable by the human eye (e.g., shape, first-order textures). The leftmost column is a zoomed-in view of the 512 × 12 pixel CT image that enters the model, representing the pre-processing done (which merely amounts to setting any pixels outside of the gross tumor volume to zero). The middle column shows gradient class activation maps (Grad-CAMs^39^) on the penultimate convolutional block, which depict what areas of the image the CNN found most relevant for outcome prediction. The heat map represents how important each region of the image is to the given classification. This information can potentially be used by clinicians to make further hypotheses regarding the nature of the tumor. The final column depicts a merger of the Grad-CAM and the CT image. This is the image we recommend is used when attempting to understand the network’s behavior on a particular input image.

**Figure 2:**
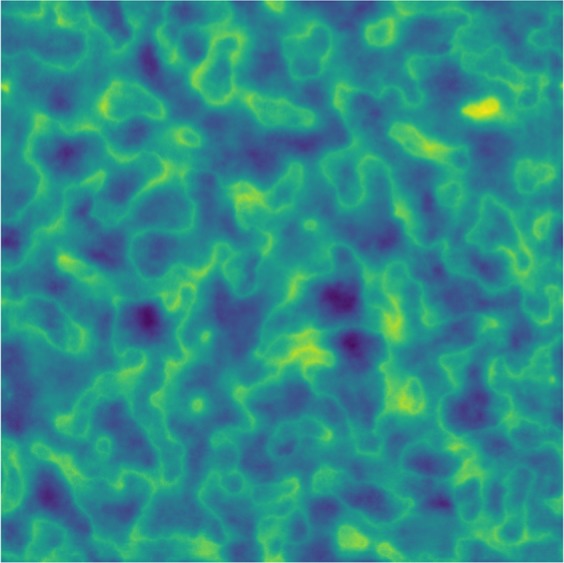
Maximal activation map depicting a procedurally generated image that results in a classification of maximal probability. Represents a procedurally generated image input that would result in a maximal classification score of 1 (i.e. distant metastasis). Of particular interest is the large scale homogeneity and the small scale heterogeneity. Color map chosen solely for visualization purposes. The maximal activation map was generated as a 512 × 512 image to spatially represent the input CT shape.

Another method of visualizing our network is through an activation map. Shown in Fig. 2, an activation map represents a procedurally generated image that would result in a distant metatasis classification of maximal probability (a score of 1). We stress that this image was generated on the fully trained network and thus does not represent an individual CT image within the dataset. The image appears quite disorderly at first, but there are some interesting aspects that we can discern from it. Firstly, the image appears mostly homogeneous on a large scale, meaning no region of the image favors one pattern over another. This is an indication that our network is approaching location invariance, due to tumor locations being highly variable regardless of outcome within the training set. Secondly, when focusing on a small portion of the image, the image appears heterogeneous both in shape and intensity. This is indicative of heterogeneous tumors being more aggressive and likely to be assigned a poor outcome, an observation consistent with the published literature^40–43^. The corresponding minimal activation map (i.e., a score of 0) is shown and discussed in the Supplementary Note/Fig. A1. Although Fig. 2 does not directly explain any specific patient prediction, it gives insight into the trained network and the patterns it is associating with a specific classification.

### Filters within the CNN explicitly recognize radiomic features

**Figure 3:**
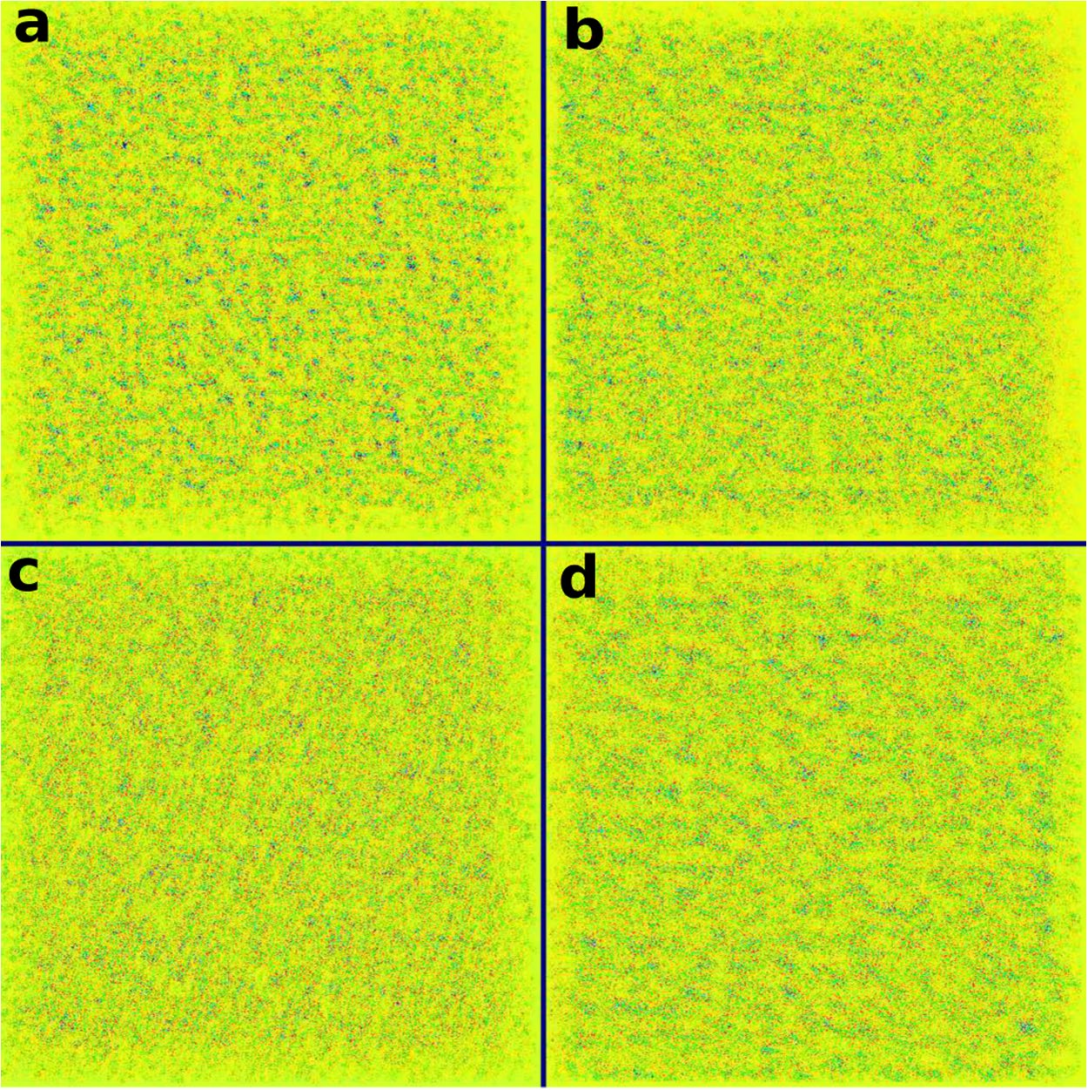
Maximal activation maps of four filters within the final convolutional layer. Represents procedurally generated images that would each result in a particular filter being maximally activated. While humans are capable of distinguishing between these four images, we are currently unable to directly interpret them. Our framework is capable of analyzing the type of data that these images represent. The lettering scheme is relevant to Fig. 4. Color map chosen solely for visualization purposes.

**Figure 4:**
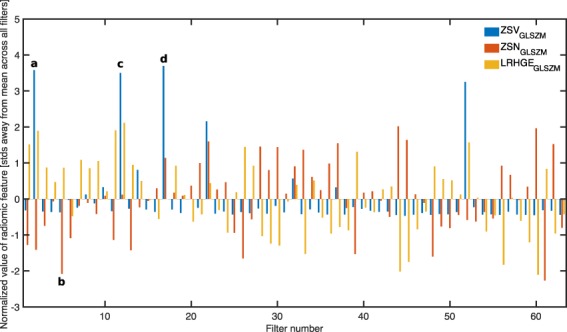
Normalized value of three radiomic features of interest across 64 convolutional filters within the final convolutional layer. *x*-axis represents which filter within the third convolutional block. *y*-axis represents the value of the radiomic feature, normalized across all filters. Blue bars represent *ZSV*_*GLSZM*_, red bars represent *ZSN*_*GLSZM*_ and yellow bars represent *LRHGE*_*GLRLM*_. Of particular interest are the numerous blue peaks (**a**, **c** and **d**). These filters are strongly activated by an input region with a high value of the radiomic feature *ZSV*_*GLSZM*_, precisely the feature that Vallières *et al*.^25^ found to be most predictive. Many of these filters are also strongly activated by extreme (high or low) values of *ZSN*_*GLSZM*_ and *LRHGE*_*GLRLM*_ (red and yellow respectively). In particular, many filters represent various permutations of the three features. As an example, (**a**) is activated by all three radiomic features, (**b**) is mostly activated by red, (**c**) is mostly activated by blue and yellow, while (**d**) is mostly activated by blue. The lettering scheme corresponds to the maximal activation maps shown in Fig. 3.

To determine whether the CNN trained *de novo* could be recognizing radiomic features, we visualized a montage of *filters*. Each convolutional block within the network functions by convolving a variable number of learned *filters* with the input data^9^. Figure 3 represents 4 of the 128 filters that make up the final convolutional block. Similar to Figs 2 and 3 is not representative of any specific CT image but rather the final trained network. Each square represents the procedurally generated input image that would maximize the mean output of a specific filter, thus informing us at an abstract level what sort of image each filter is interested in when making a decision. It is evident that each of these filters is maximally activated by various *textures*, rather than a particular shape or object as is common in more typical convolutional neural networks. In order to determine whether any of the filters were specifically activated by previously engineered radiomic features, we extracted 94 radiomic features (as described in the Image Biomarker Standardization Initiative (IBSI^29^)) from each filter’s maximal activation map (Fig. 3). An example of this analysis when performed on 64 of the filters (chosen among those whose maximal activation maps converged) is shown in Fig. 4. The *y*-axis represents the normalized value of a specific radiomic feature that Vallières *et al*.^25^ found to be predictive (blue: *ZSV*_*GLSZM*_, red: *ZSN*_*GLSZM*_, yellow: *LRHGE*_*GLRLM*_). These features were calculated using the exact same extraction parameters as the benchmark study. The letters indicate the corresponding square in Fig. 3. Of particular interest are the numerous blue peaks (a,c and d). These filters are strongly activated by an input region with a high value of the radiomic feature *ZSV*_*GLSZM*_, precisely the feature that Vallières *et al*.^25^ found to be most predictive. Many of these filters are also strongly activated by extreme (high or low) values of *ZSN*_*GLSZM*_ and *LRHGE*_*GLRLM*_ (red and yellow respectively). In particular, many filters represent various permutations of the three features. As an example, (a) is activated by all three radiomic features, (b) is mostly activated by red, (c) is mostly activated by blue and yellow, while (d) is mostly activated by blue. In essence, these filters are recognizing and combining various radiomic features to help classify a particular input image.

The 64 filters’ maximal activation maps are displayed in Supplementary Fig. A2. The radiomic analysis for these 64 filters and all 94 radiomic features (as described in the Image Biomarker Standardization Initiative (IBSI^29^) and extracted using 128 gray levels and a scale of 1 mm) is displayed in Supplementary Fig. A3.

## Discussion

These results show great potential in using convolutional neural networks trained *de novo* on medical gray-scale images to predict oncological treatment outcomes. The average adult human is capable of looking at an object or person and immediately classifying it properly (type of object/name) with virtually 100% accuracy. This is largely due to the types of features that our brains have developed to subconsciously look for and associate with a particular object or person (e.g. sharp edges/color gradients combining into shapes). These concepts are precisely what networks trained on ImageNet have learned to process. A major benefit to training a network *de novo* is that it can learn abstract concepts unique to the dataset of interest such as specific radiomic texture features. This is explicitly shown in Figs 3 and 4. The radiomic analysis performed (Fig. 4) shows that many of these filters are able to process and distinguish radiomic features *without explicitly being told the definition of any feature*. The primary example of this are the numerous blue peaks in Fig. 4. Effectively, these filters have learned the ability to see an image from a perspective that is interested in the value of the radiomic feature *ZSV*_*GLS*′*ZM*_. Furthermore, this feature is one of the radiomic features that Vallières *et al*.^25^ found to be predictive. Similarly, many of the filters are strongly attuned to the features *LRHGE*_*GLRLM*_ and *ZSN*_*GLSZM*_, the two other features that Vallières *et al*.^25^ found predictive. The ability of our network to directly recognize radiomic features without being told their definition is powerful for a number of reasons, one of which being it may remove the need to specifically engineer new features. The relationship between the filters of our network and the most predictive radiomic features of the compared study also helps build confidence in the network’s output. As seen in Table 1, a combination approach does increase the AUC from 0.88 to 0.92. This indicates that although our network does recognize the features to some extent, it does not directly represent them. In other words, there is still some orthogonality between the quantitative value of a radiomic feature and the filter’s impact on our network’s output score. Future work could be done to further investigate the difference between the two representations.

The visualization tools we used in this work begin to overcome one of the primary obstacles in outcome analysis using a machine learning model: interpretability. The benchmark study found the most predictive combination of radiomic features related with distant metastasis to be *LRHGE*_*GLRLM*_, *ZSV*_*GLSZM*_ and *ZSN*_*GLSZM*_. While these features are mathematically well-defined, it is difficult for humans to visualize them, let alone develop an intuition for them. In comparison, the visualization tools our framework uses, particularly the class activation maps (Fig. 1), are more interpretable. It is noted that the interpretations discussed thus far do not exhaust all the information our method can extract. Through future research and collaboration, it is our belief that our framework can lead to further hypotheses. The visualization tools grant the ability to not only have more confidence in the output, but also provide a tool to the medical community that may help discover unknown aspects of these gray-scale images.

One major advantage of this framework is the lack of feature engineering, in stark contrast to traditional radiomic frameworks. In particular, the benchmark study^25^ required the extraction of 55 pre-defined radiomic features, 40 of which were extracted using combinations of three parameters (isotropic voxel size, quantization algorithm and number of gray levels). In total, this resulted in the extraction of 1615 radiomic features. Prior to the extraction, an elaborate and complex feature selection process was required to identify the “potentially useful” features^25^. Elaborate procedures for the selection of potentially useful features have also been developed for radiomic analyses of other cancer types^44^. The approach developed in this report eschews this problem by giving the algorithm the full set of un-altered pixel data of the tumor and allowing the algorithm to tell the user what is important rather than the user explicitly telling the algorithm what is important. This is one of the primary motivations behind our exploration of a end-to-end CNN, without any feature or machine learning algorithm selection. Similarly, as the handcrafted features were extracted from pre-segmented tumors, maintaining this segmentation allows us to better evaluate the hypothesized connection between the CNN’s behavior and the radiomic features.

There are improvements that could be made to this framework to potentially increase prediction performance and generalizablity. As an example of a potential pre-processing step, the CT images could be cropped to include only the field-of-view surrounding the tumor itself. This could improve the learning capabilities of the algorithm particularly by improving the location invariance of the model. However, this would be (albeit slightly) complicating the framework, directly opposing one of its primary advantages. There are two sources of information that could be added to our framework. The first would be to fully incorporate 3-dimensional information. In contrast to the radiomic study^25^ which used the entire tumor volume, our framework only considers the central slice (with robustness estimated by training on one slice superior/inferior). It is emphasized that this study was capable of surpassing the predictive power of the benchmark study solely using a single 2-dimensional image. By incorporating the entire tumor, the performance could potentially be further improved. However, convolutional neural networks which incorporate 3-dimensional image information are architecturally complex and computationally expensive. Another information source is each patient’s positron emission tomography (PET) image. Vallières *et al*.^25^ found additional predictive power in each patient’s PET image, so incorporating this information into our framework should improve performance. Potential image manipulation needs aside, this would be computationally simpler than incorporating volumetric information. The CT + PET image could be introduced into the network in a 2-channel fashion: input data would be 512 × 512 × 2 pixels, rather than 512 × 512 × 1 pixels. This is akin to the 3-channel RGB input that many traditional CNNs use. Additionally, our framework uses solely the pre-segmented gross tumor volume as an input rather than including the surrounding tissue. Ideally one would include the surrounding tissue and build a network capable of incorporating any information within. This would also remove the need for location invariance, as there could be additional information contained in the tumor’s precise location within the anatomy. Finally, while we performed a relatively simple combination approach (logistic regression), future work could study the different methods that could be used to better combine traditional radiomics and CNN information. An in-depth study regarding transfer learning and whether a transfer-learnt network is capable of recognizing the same radiomic features could also be performed. These improvements were not included to align with the goal of assessing our primary hypothesis, keeping the initial framework as simple as possible and to reduce training time.

This report showed the power and potential of using a deep convolutional neural network built *de novo* to perform outcome predictions on the pre-treatment CT image of head and neck cancer patients. Often transfer learning is used to train a CNN on a new dataset due to the perception that thousands, if not millions of images are required to build an accurate model. Our framework shows that a training set of 200 medical gray-scale images may be sufficient to train a network *de novo*, with proper data augmentation. The model was shown to have the ability to explicitly recognize radiomic features and further improve on the performance of a traditional radiomics framework. Performance gains aside, our framework overcomes many of the typical issues when building a traditional radiomics-based model. Specifically, our model is capable of being interpreted in a more intuitive fashion and completely eschews the need for feature engineering. We believe our framework could serve as the base of a gray-scale image analysis tool capable of being adapted to other imaging modalities (e.g., PET, MRI) or other cancer sites (e.g., liver, lung, breast).

## Methods

### Patient cohorts

Extensive details regarding the patient cohort used throughout this report are publicly available on The Cancer Imaging Archive (TCIA)^25,45^ repository. Eligible patients were taken from four separate institutions (Hôpital général juif (HGJ), Centre hospitalier universitaire de Sherbooke (CHUS), Hôpital Maisonneuve-Rosemont (HMR) and Centre hospitalier de l’Université de Montréal (CHUM)). The majority received chemotherapy adjuvant to radiotherapy (92%) while the remainder solely received radiation (8%). All patients underwent joint FDG-PET/CT scans, however this study only made use of the CT image. The training set was defined as the patients from HGJ and CHUS, while the validation set was defined as the patients from HMR and CHUM. This was the same distribution used in the compared study^25^, specifically notable due to the validation set only containing patients from independent institutions. Any patients with metastases or recurrent H&N cancer at presentation were excluded, along with any patients receiving palliative care. The median age of patients across the total cohort was 63 years (range: 18–88). The median follow-up period across all patients was 43 months (range: 6–112). Any patients that did not develop cancer recurrence and had a follow-up period of less than 24 months were discarded. The outcome distribution for both cohorts is shown in Table 3. It is noted that 2 patients from the training cohort were lost to data corruption.

**Table 3:** Patient/outcome distribution^25,45^.

	Training cohort	Validation cohort
Total	194	106
Outcome		
Distant metastasis (DM)	26 (13%)	14 (13%)
Loco-regional failure (LRF)	29 (15%)	16 (15%)
Death	32 (16%)	24 (23%)
Institution		
Hôpital général juif (HGJ)	92 (47%)	—
Centre hospitalier universitaire de Sherbooke (CHUS)	102 (53%)	—
Hôpital Maisonneuve-Rosemont (HMR)	—	41 (39%)
Centre hospitalier de l’Université de Montréal (CHUM)	—	65 (61%)
Tumor type		
Oropharynx	129 (66%)	77 (73%)
Hypopharynx	5 (3%)	7 (7%)
Nasopharynx	20 (10%)	8 (7%)
Larynx	36 (19%)	9 (8%)
Unknown	4 (2%)	5 (5%)

### Convolutional neural network architecture

Our CNN contains four main operations: convolution, non-linearity, pooling and classification. These four operations are facilitated by *layers*, which are the building blocks of the overall framework. The convolution operation is ultimately what learns and subsequently extracts features from the input data. The layer includes a variable number of *convolutional filters*, each of which acts as a sliding window (of a small size, e.g., 5 × 5 pixels) applying a convolution over the input data. By learning a number of different filters (e.g., 64), the network is able to incorporate a large variety of features. The more filters we choose to learn, the more image features the network is able to ultimately extract and recognize in unseen images. The non-linearity operation is needed to accurately model the type of real-world data we are interested in. Many CNNs have adopted the use of a rectified linear unit (ReLU), which simply replaces all negative input values (from the preceding convolutional layer) by 0. Instead, we use a parametrized rectified linear unit (PReLU), which has largely the same effect but allows a small amount of the negative input values to propagate through the network by multiplying the negative portion of the input domain by a learnt non-zero slope^46^. Next, the pooling operation serves to progressively reduce the spatial size of the input information. This is important for computational efficiency, to ensure that the model can be generalized and most importantly, to introduce location invariance. In our model we utilize “max-pooling”, an operation that replaces every 4 × 4 region of input data with the maximum value among them. Finally, the classification operation takes all the high-level features from the previous representations and combines them using a sigmoid activation function to determine which class the input represents.

Henceforth, we will refer to *convolutional blocks*, which contain the convolution operation, the non-linearity operation and the pooling operation stacked one after another. By stacking convolutional blocks, the network is able to progressively learn complex image features that humans are not used to processing.

**Figure 5:**
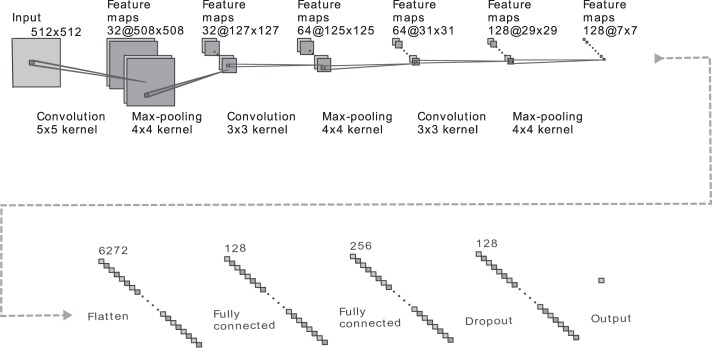
Depiction of our convolutional neural network’s architecture. Text below the graphic represents the operation between layers. Text above the graphic represents the number of feature maps or nodes within the layer. The CNN consists of three consecutive *convolutional blocks*, each of which contain a convolutional layer (of varying filter size), a max-pooling layer (4 × 4 kernel) and a parametric rectified linear unit (not shown). Following this, the output is flattened and proceeds through two fully connected layers, a parametric rectified linear unit (not shown) and a dropout layer prior to being classified via a sigmoid activation function.

Our CNN architecture is shown in Fig. 5 and was largely chosen for its simplicity, a characteristic that increases the model’s ability to generalize and reduces the risk of over-fitting. Of particular note is the usage of a 512 × 512 pixel input layer, allowing any standard CT image to directly be analyzed by the network with minimal pre-processing. The input layer is followed by 3 consecutive convolutional blocks. Each block consists of a convolutional layer, a max-pooling layer and a PReLu layer. It is noted that the PReLu layers are not explicitly depicted in Fig. 5. The convolutional layers used a filter size of 5 × 5 pixels, 3 × 3 pixels and 3 × 3 pixels, respectively. These layers formed the foundation of the network, each subsequent layer uncovering more complex features. The first block containing a larger filter allowed the later layers to have larger effective fields of view, combining a larger number of input pixels to determine important features. Notably, the max-pooling layers grant the network some degree of location invariance, a crucial attribute given the fact that the location of the tumor within the CT should not impact the outcome. The 3 convolutional blocks were followed by two consecutive fully connected layers and a final PReLu. Finally, a drop-out layer was included after the last fully connected layer, directly prior to the classification layer. Drop-out played a major role in reducing over-fitting by removing half of the information every single stochastic gradient descent iteration. Each iteration, the output of half of the nodes in the final fully connected layer were set to 0. This teaches the network that it must be capable of functioning even with a substantial amount of missing information, effectively forcing it to not rely too heavily on a single piece of information^9^.

### Implementation details

Our framework was built on Python3 using the Keras library operating on the well-optimized tensor manipulation library Tensorflow^47,48^. The final outcome probability (in the classification layer) was computed using a sigmoid classifier. Each convolutional block used a PReLU as an activation function. The network weights were optimized using a stochastic gradient descent algorithm with a fixed learning rate of 0.001 and a momentum of 0.5. The mini-batch size was 32 and the objective function used was binary cross-entropy. Image augmentation was performed to increase generalization and reduce the training bias that the network is inherently subjected to^9^. Prior to training, each image was randomly flipped (horizontally and/or vertically), rotated a random amount (0–20°), and shifted a random fraction (0 to 0.4 times the total width of the image) in a random direction. This resulted in the total training dataset (and thus a single epoch) consisting of 4000 images (each tumor is augmented roughly 20 times). Our algorithm was trained and evaluated on a pair of NVIDIA GTX 1080TI graphic processing units to exploit their computational speed. Total training time for one network required approximately 5 hours (100 epochs). The time required to predict outcomes on the validation cohort is approximately 100 milliseconds. More details regarding the implementation and the specific range of parameters tested can be found in the Supplementary Methods.

### Code availability

Code to build, compile, train and evaluate each model along with all visualization scripts will be publicly available on Github.

## Supplementary information

Supplementary Information

## Data Availability

The dataset analyzed throughout this study is publicly available on The Cancer Imaging Archive (TCIA) repository (10.7937/K9/TCIA.2017.8oje5q00)^25,45,49^.
