# Supplementary material for: Deep learning in head & neck cancer outcome prediction
Source: Sci Rep. 2019 Feb 26;9:2764. doi: 10.1038/s41598-019-39206-1 (PMC6391436; doi:10.1038/s41598-019-39206-1)
Supplement: Supplementary file 1 — Supplementary Information [file 41598_2019_39206_MOESM1_ESM.pdf]

Deep learning in head & neck cancer outcome prediction

Supplementary Information

André Diamant, Avishek Chatterjee, Martin Vallières, George Shenouda, Jan Seuntjens

Supplementary Note 1: Minimal activation map

The procedurally generated minimal activation map is shown in Figure A1. Evidently, this figure represents noise. This is expected as the algorithm is trained to evaluate whether an image is likely to be an aggressive tumor. Thus, procedurally generating an image to which the network would assign a *minimal* score ends up resembling noise. This further reinforces that the maximal class activation map (and its properties) represent a *difference* between more and less aggressive tumors.

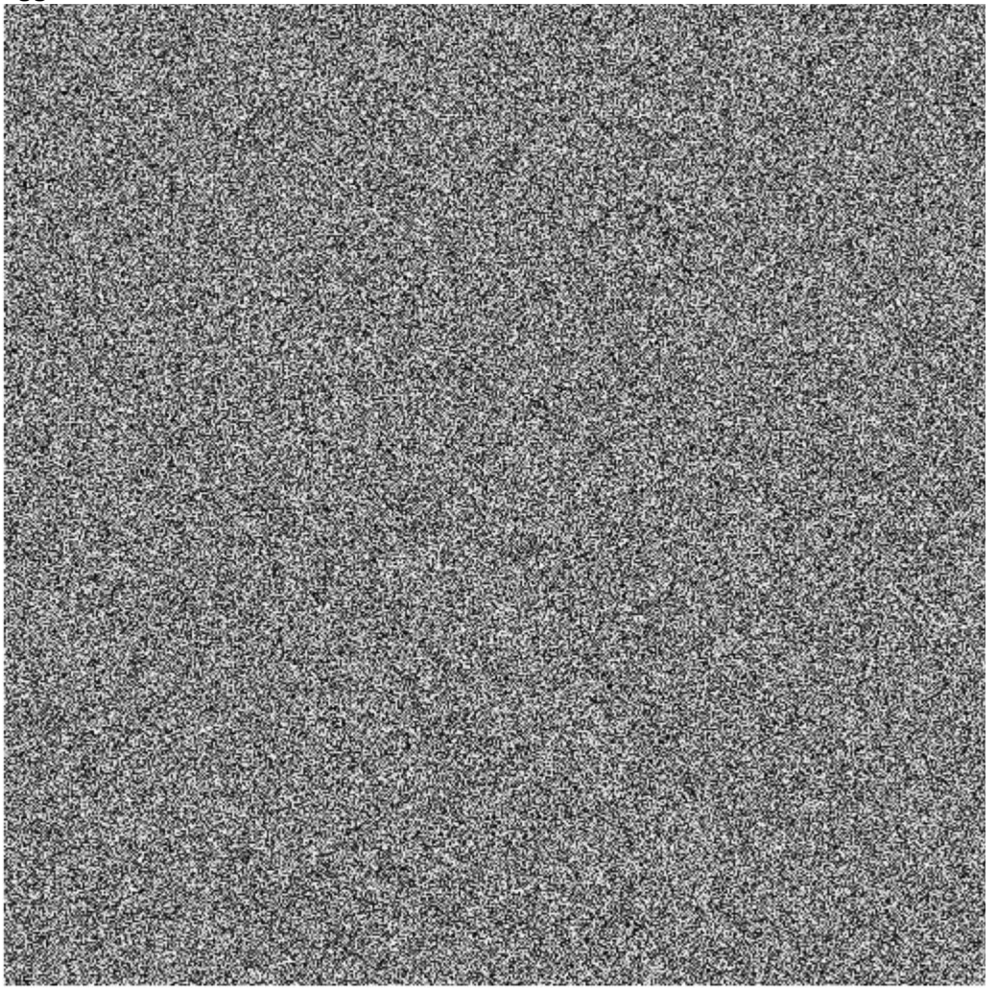

Figure A1: ***Minimal activation map****. Depicts a procedurally generated image input that would result in a minimal classification score of 0 (i.e. not distant metastasis).*

Supplementary Note 2: Radiomic analysis

As mentioned in the manuscript, the radiomic analysis (for 94 radiomic features as described in IBSI[1]) was performed for the top 64 filters that make up the final convolutional block. The maximal activation maps for the 64 filters are shown in Figure A2 (low-resolution version, high-

resolution version requires separate download due to size). The radiomic analysis results are shown in Figure A3.

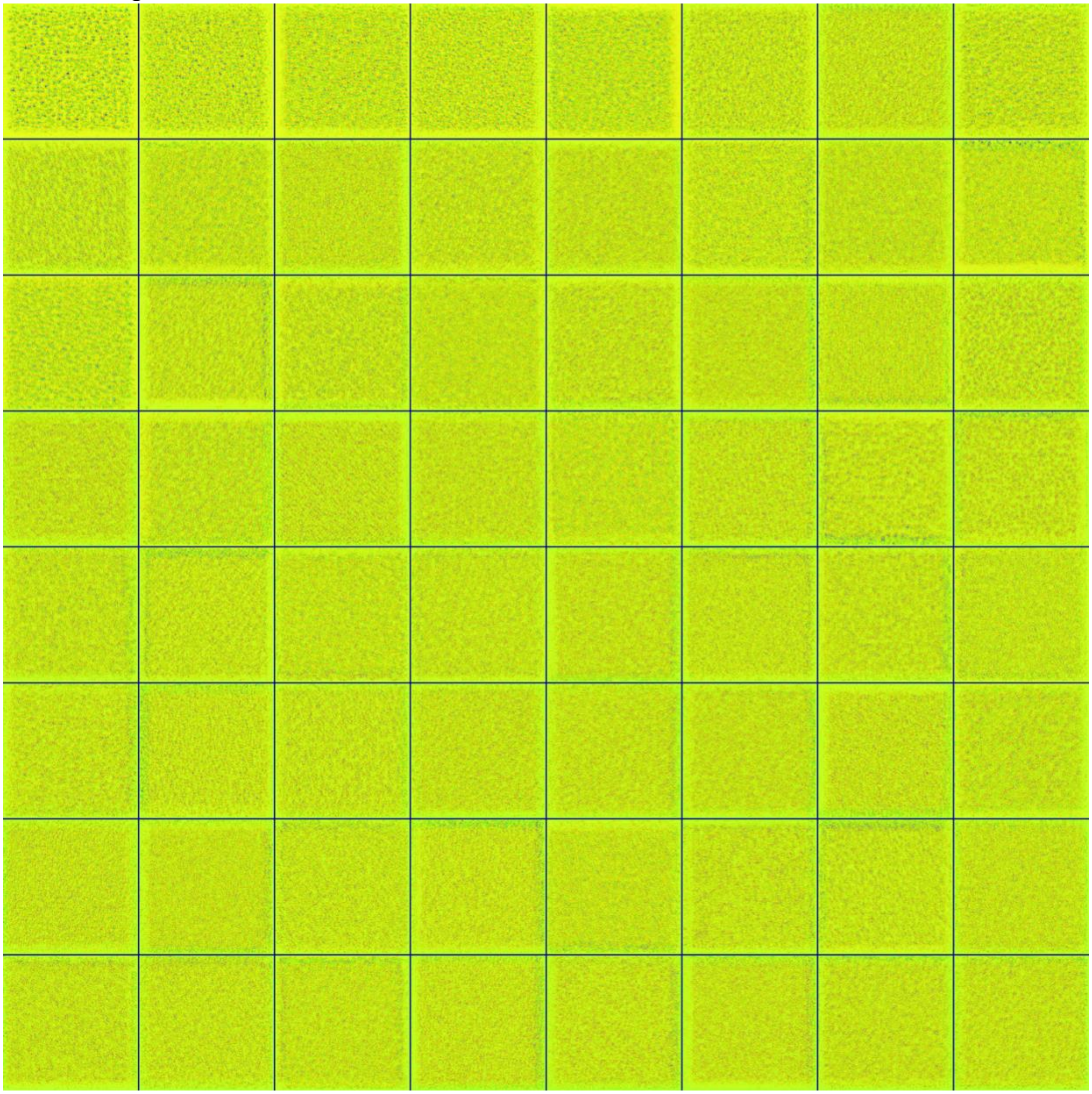

*Figure A2: **Maximal activation maps for top 64 filters in the final convolutional block**. Represents procedurally generated images that would each result in a particular filter being maximally activated. Color map chosen solely for visualization purposes.*

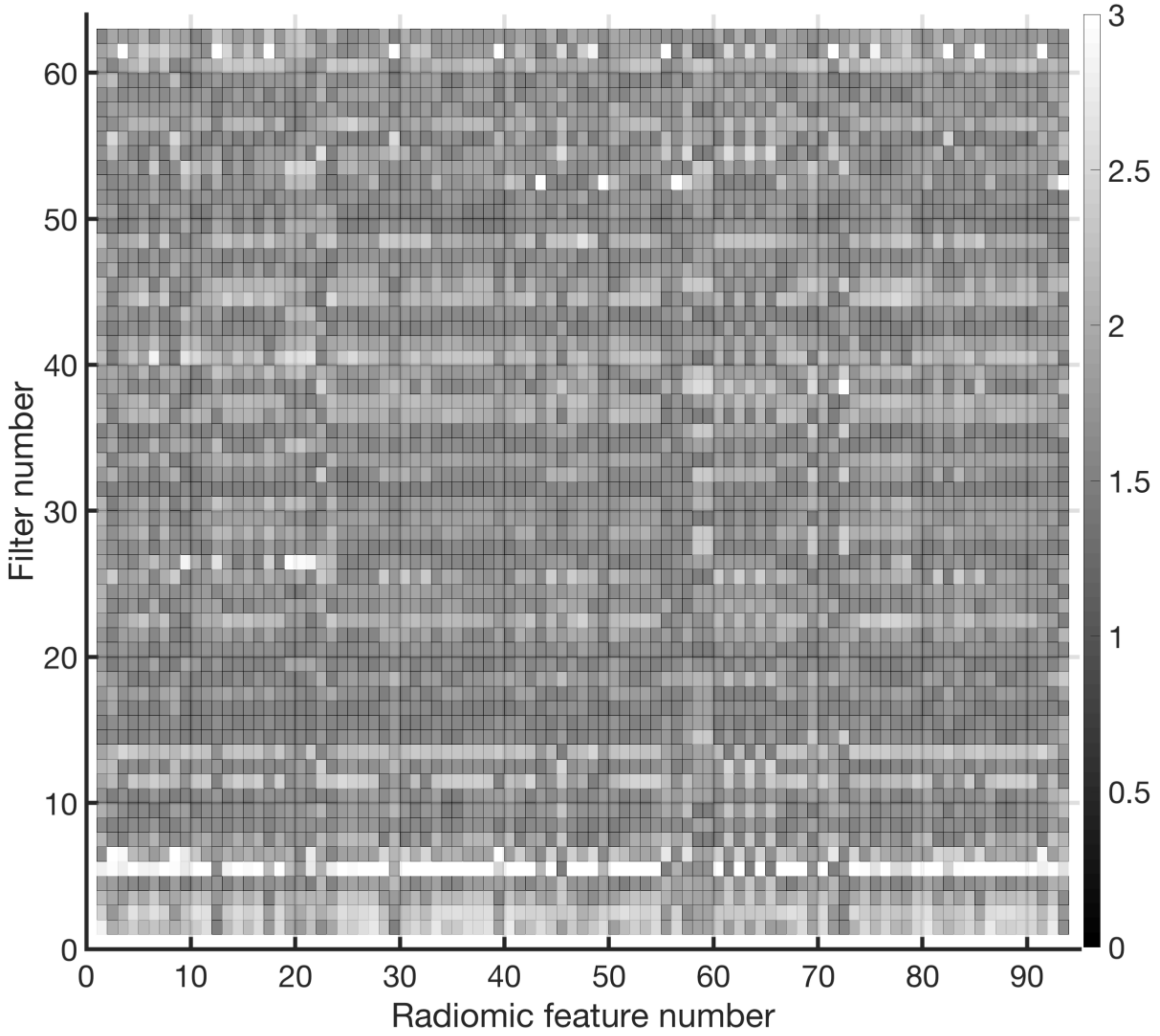

Figure A3: ***Radiomic analysis across all features and filters****. x-axis represents the radiomic feature number as defined in the Image Biomarker Standardization Initiative (IBSI). y-axis represents the filter number (arbitrary). Color indicates the normalized value of the radiomic feature (standard deviations away from the mean calculated for a specific feature across all filters). Color scale chosen solely for visualization purposes. This analysis represents that the range of filters represents a diverse selection of radiomic features, combined in a number of different ways.*

Supplementary Methods 1: Transfer learning approach

VGG19 was used as a transfer learning base, chosen for being the most recently successful network to win ImageNet while still maintaining similar architecture to ours (in comparison to the more advanced ResNet/GoogleNet). More explicitly, VGG19's final layers involve multiple fully connected layers, concluding in a fully connected layer with *x* nodes, where *x* is the number of image classes. This is in comparison to more advanced networks, which may not employ the same fully connected layer stack. Three approaches were taken. Firstly, the convolutional base (and weights) of VGG19 were used with a randomly initialized fully connected stack identical to the bottom portion of Figure 5. This fully connected stack was subsequently fine-tuned, with a learning rate of 10^{-4}, keeping the convolutional base weights

frozen. As VGG19 requires images of shape 224 X 224, our images had to be cropped to this size. This was done while keeping the GTV itself as centered as possible in the cropped image. After 100 epochs of training using the same partition scheme as the benchmark study, this resulted in an AUC of 0.69 predicting DM. The second method used an identical architecture, except the fully connected stack was not randomly initialized, but rather used the learnt weights from our top performing model. It was subsequently fine-tuned for 100 epochs, using a learning rate 10^{-4}. This resulted in an AUC of 0.72. The final approach was identical to the second approach; however, we only trained the final fully connected layer. That is, only trained 4096 parameters. This resulted in an AUC of 0.76, slightly better when compared to the previous transfer learning methods utilized, however still substantially worse than our *de novo* methodology. More work is needed to investigate the full implications of using transfer learning in this context; for our study we further concentrated on the performance of our own CNN constructed for this purpose.

Supplementary Methods 2: Parameters tested when building the CNNs

A variety of architectures and parameters were tested during the course of this research. A summary of what values were tested is shown in Table 1. A more detailed explanation of each parameter and the range tested is described below[2]. We note that the exact choice of architecture and parameters is a result of an educated trial-and-error process, and the intuition gleaned from it. There is a decent degree of robustness in the variation of many parameters (e.g. size of fully connected layers, number of filters, type of non-linearity), as slight modifications will not qualitatively alter the results.

Table 1: Summary of parameters tested during the course of this research. Bolded value represents the value(s) settled on in the final model.

PARAMETER TESTED	VALUES TESTED
**BATCH SIZE**	16, **32**, 48
**NUMBER OF FILTERS**	**32**, **64**, **128**, 256, 512
**NUMBER OF CONVOLUTIONAL BLOCKS**	2, **3**, 4
**TYPE OF NON-LINEARITY**	**PReLU**, ReLU
**FILTER SIZE**	**3x3**, **5x5**, 7x7, 9x9, 12x12
**TYPE OF POOLING**	**Max pooling**, Average pooling
**SIZE OF POOLING**	2x2, **4x4**, 6x6
**NUMBER OF FULLY CONNECTED LAYERS**	1, **2**, 3
**SIZE OF FULLY CONNECTED LAYERS**	64, **128**, **256**, 512
**LEARNING RATE**	10^{-2}, $\mathbf{10^{-3}}$, 10^{-4}, 10^{-5}
**MOMENTUM**	0.25, **0.5**, 0.75, 0.9
**ROTATION RANGE (DATA AUGMENTATION)**	$\mathbf{20^o}$, 30°, 40°
**SHIFT RANGE (DATA AUGMENTATION)**	0.2, 0.3, **0.4**

Batch size: The batch size represents the number of images that the network sees each iteration of the algorithm. One *epoch* consists of several batches such that the network sees every image once (i.e. *# of batches/epoch = total # of images / batch size)*. The performance seemingly began to plateau at the chosen batch size (32), higher batch sizes did not improve the performance. Furthermore, at higher batch sizes, computational memory becomes a serious concern, limiting our possible range of batch sizes.

Number of filters: The number of filters represents the number of learned weight matrices within each convolutional layer. A variety of permutations was tested, always with deeper layers including either an equal number or more (often double) filters than the previous layer. The range (from 2^5 to 2^9) is a typical range tested when building a CNN framework. Our network leans towards using a smaller number of filters when possible, to avoid over-fitting.

Number of convolutional blocks: The number of convolutional blocks drastically impacts the complexity of the network. CNNs are prone to over-fitting, especially with a larger number of convolutional blocks, limiting our range to that shown above. In particular, the CNN began to overfit when using 4 convolutional blocks as opposed to 3.

Type of non-linearity: Parametric rectified linear units (PReLU) introduce a minor amount of complexity into the network, which in our case resulted in a performance increase of approximately 2%.

Filter size: Filter size represents the effective impact that a single pixel has on deeper layers. A particularly large range was chosen as *a priori* the relevant neighbourhood around any given pixel is unknown. As most tumors within our dataset were relatively small, the larger filter sizes (i.e. 9 x 9 or higher) were quickly dismissed.

Type of pooling: Average pooling was attempted, but quickly dismissed for generalization purposes. In our case, max pooling results in a network with significantly less over-fitting. This is as expected, as each max-pooling layer effectively reduces the amount of information (which possibly could be used to over-fit) by nearly 95%. In comparison, average-pooling reduces the dimensionality, but still allows much of the information to flow through to the next layer.

Size of pooling: Larger max pooling results in less over-fitting, but 6x6 resulted in the loss of a substantial amount of information (particularly with the filter size used).

Number of fully connected layers: The number of fully connected layers significantly impacts the complexity of the network. It also exponentially increases the computational power required, restricting the maximum range. Like the number of convolutional blocks, we found the network began to overfit when using more than 2 fully connected layers. On the other hand, using 2 fully connected layers instead of 1 resulted in a significant performance increase (on the order of 10%).

Size of fully connected layers: Similarly, the size drastically impacts the complexity and the computational power required. It is typical to test up to a size of 2^{10}, however we did not due to computational reasons.

Learning rate: Learning rate affects the step size that the algorithm takes during the gradient descent algorithm. A learning rate that is too high can skip over a local/global minimum, while a learning rate that is too low can get caught in a local minimum. We also experimented with lowering the learning rate once the algorithm was close to a global minimum, however it did not result in a performance gain.

Momentum: Similar to varying the learning rate, momentum can help the algorithm not get caught in a local minimum. It does this by not taking a step solely based on the gradient, but also incorporates the current velocity (akin to classical momentum). A wide range was tested as *a priori* it is difficult to know what the proper value for our particular network is.

Rotation range (data augmentation): The rotation range was chosen to enable most possible arrangements of a particular tumor (as each image was also flipped horizontally and/or vertically).

Shift range (data augmentation): The shift range was chosen as to not create a situation where a tumor could be shifted entirely out of frame (<1% chance even at the maximum shift value).

Number of epochs: To ensure that each network had enough training time, they were trained for 400 epochs and the training/validation loss was visually inspected. An example of the training (blue)/validation (orange) loss progress for is shown in Supplementary Figure A4. At approximately 100 epochs, the network began to over-fit the training data (as shown by the steeper slope on the training curve and drastic increase in the validation loss. Ultimately, the model reached its validation loss minimum at the 34th epoch.

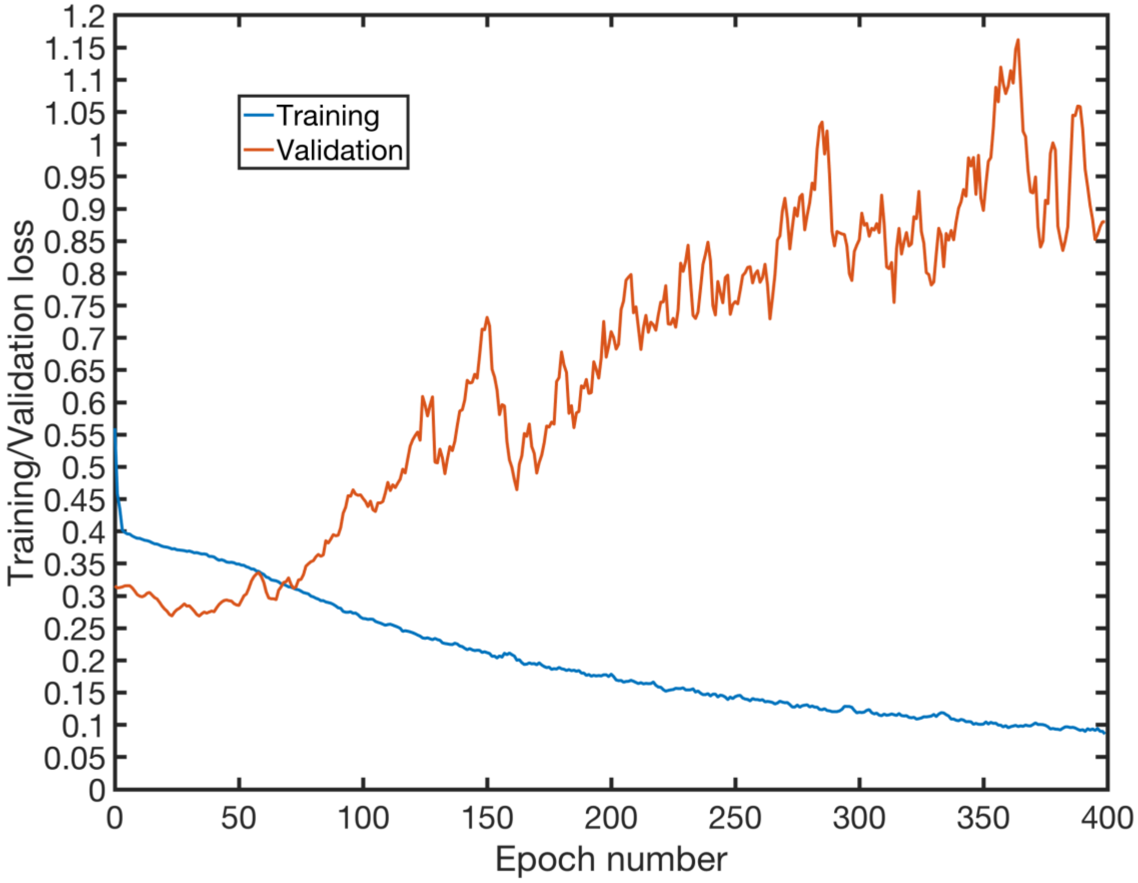

*Figure A4: **Training/Validation loss for a sample training of a network**. Orange represents the loss measured on the validation data-set, blue represents the loss measured on the training data-set.*

Supplementary References:

1. Zwanenburg, A., Leger, S., Vallières, M. & Löck, S. Image biomarker standardisation initiative. *Arxiv* (2016). doi:https://arxiv.org/pdf/1612.07003.pdf
2. Goodfellow, I., Bengio, Y. & Courville, A. *Deep Learning*. (MIT Press, 2016). doi:10.1038/nmeth.3707
